# Comparative Assessment of Three Medicinal Plants against Diabetes and Oxidative Stress Using Experimental and Computational Approaches

**DOI:** 10.1155/2023/6359818

**Published:** 2023-04-25

**Authors:** Md. Shahin Shah, Md. Saddam Hossain Talukder, A. M. Kafil Uddin, Muhammad Nazmul Hasan, Syed Al Jawad Sayem, Gomaa Mostafa-Hedeab, Md. Masudur Rahman, Rohit Sharma, Ayman A. Swelum, Amany Abdel-Rahman Mohamed, Talha Bin Emran

**Affiliations:** ^1^Department of Pharmacy, International Islamic University Chittagong, Chittagong 4318, Bangladesh; ^2^Pharmacology Department & Health Research Unit, Medical College, Jouf University, Sakakah, Saudi Arabia; ^3^Department of Rasa Shastra and Bhaishajya Kalpana, Faculty of Ayurveda, Institute of Medical Sciences, Banaras Hindu University, Varanasi 221005, Uttar Pradesh, India; ^4^Department of Animal Production, College of Food and Agriculture Sciences, King Saud University, Riyadh 11451, Saudi Arabia; ^5^Department of Theriogenology, Faculty of Veterinary Medicine, Zagazig University, Zagazig 44511, Egypt; ^6^Department of Forensic Medicine and Toxicology, Zagazig University, Zagazig 44511, Egypt; ^7^Department of Pharmacy, BGC Trust University Bangladesh, Chittagong 4381, Bangladesh; ^8^Department of Pharmacy, Faculty of Allied Health Sciences, Daffodil International University, Dhaka 1207, Bangladesh

## Abstract

The hilly and rural areas' people of Bangladesh have a great history of putting into use numerous traditional medicinal plants to cure diseases. Therefore, with ethanol extract of *Molineria capitulata* (EEMC), methanol extract of *Trichosanthes tricuspidata* (METT), and methanol extract of *Amorphophallus campanulatus* (MEAC), we mandate evaluation of in vitro *α*-amylase inhibition, antioxidants, and molecular docking, and ADMET/T analysis. According to iodine starch methods, *α*-amylase inhibition was performed, and quantitative total phenolic and flavonoid content was determined by established methods, whereas DPPH free radical scavenging and reducing power assays were performed in previously established protocols, respectively. A comparative study among three plants (EEMC, METT, and MEAC) possessed a significant (*p* < 0.01) effect but EEMC showed the highest impact on enzyme inhibition. Plants in the measuring phenolic content METT and flavonoid measurement MEAC displayed most potent in the same way in the DPPH test was METT, and in reducing power capability MEAC has showed the highest effect between three extracts. Docking's study also reveals the compounds of METT (Cyclotricuspidoside A and Cyclotricuspidoside C) exhibit the superior score among all the compounds. This finding indicates that EEMC, METT, and MEAC substantially impact *α*-amylase inhibition along with antioxidants. *In silico* study also reveals the potency of these plants, but further in-depth, precise molecular studies are needed.

## 1. Introduction

Diabetes has been affecting countless people over the world, which is due to starch, fat, and protein metabolic upset [1]. Progressing estimations exhibit that nearly 2.8% of the total population has diabetes and this number will reach 5.4% by 2025 [2]. We may face a worldwide type 2 diabetes disorder pandemic within the following 20 years. Even though new diabetic disorders the entirety relies on the glucose models used to symbolize diabetes, the rate and predominance of type 2 diabetes have been expanding [3]. Diabetics additionally seem to build the creation of professional provocative cytokines and incendiary arbiters, for example, interleukin-1 (IL-1), interleukin-6 (IL-6), tumor rot factor-*α*(TNF-*α*), macrophage chemoprotectant-1 (MCP-1), and nitric oxide (NO) which are likewise connected to the pathogenesis of diabetes [4]. Although some salve has been used extensively during the past few decades generally, the truth is that they have also been reflecting unforeseen scenarios. Therefore, keeping up stable and lower blood glucose can accomplish by postponing glucose assimilation through restraint of sugar hydrolyzing proteins, for example, *α*-glucosidase and *α*-amylase in the stomach-related tract [5]. The *α*-amylase (*α*-1,4-glucan-4glucanohydrolases) is an eminent secretory result of the pancreas and salivary organ liable for the underlying advance in the hydrolysis of complex sugar to a blend of oligosaccharides and disaccharides in the intestinal mucosa [6]. There are a few points of interest in common home-grown medications, for example, a decrease in the danger of reactions, the viability of interminable conditions, far-reaching accessibility, and minimal effort. Consequently, inhibitors of the *α*-amylase compound, which is separated from plants, could be developing contenders to control hyperglycemia in diabetic patients [7].

Reactive oxygen species (ROS) contain highly reactive molecules utilizing oxygen metabolism [8, 9]. ROS, such as hydroxyl radicals, superoxide radicals, peroxyl radicals, and hydrogen peroxide are constantly generated as byproducts of metabolic reactions or from several exogenous factors. They serve an important physiological function in low to moderate concentrations, such as immunocompetence, apoptosis, hormonal regulation, signal transduction, transcription factors, and adaptive responses to enzymes [4]. But an excessive production of ROS and a weakened antioxidant defense system often lead to the development of oxidative stress (OS) [5, 6]. Oxidative stress (OS) is one of the key factors in inducting a variety of chronic and degenerative diseases, including atherosclerosis, ischemic heart attack, aging, and diabetes mellitus; along with this, cancer, immunosuppression, and neurological disorder [7]. Natural antioxidants obtained from plant sources are considered a significant approach in retarding the prognosis of diabetes and other chronic diseases as they are capable of neutralizing ROS thus alleviating oxidative stress [8–10]. Secondary plant metabolites such as flavonoids and tannins are rich in antioxidant activity, which are believed to be efficient in resisting the destruction of pancreatic *β*-cells and diabetes-induced ROS production. Thus, a plant having a strong enzyme inhibitory and antioxidant potential may be considered an important therapeutic candidate for managing diabetes [11].

*Molineria capitulata* is generally a stemless, stout herb-type plant known as palm grass, which is up to 1 m in length and belongs to *Hypoxidaceae* [12, 13]. Traditionally different parts of *M*. *capitulata* are used for various purposes such as rhizomes decocted with herbal medicines for the management of consumptive cough, asthenia, impotence, and spermatorrhea. In India, it was initially recorded as a treatment for hemorrhoids, asthma, jaundice diarrhea, colic, gonorrhea, and roots and leaves used in country liquor. The contemporary investigation addressed the presence of several isolated phytoconstituents such as Crassifoside A, Breviscaside A, Crassifogenin C, Crassifoside D, Curcapital, Isocurculigine [14]. In Addition, the current study proposed the hypoglycemic and anthelminthic activities proclaimed from roots [15].

*Trichosanthes tricuspidata* is tribally known as Indrayan and makal. Morphologically it is a climber strong woody tree, with a height of 5–20 m; furthermore, it belongs to *Cucurbitaceae* [16]. Different parts of *T*. *tricuspidata* have different ethnomedicinal effects, such as fruits for asthma, carminatives, leprosy, and rheumatism. Furthermore, seeds have emetic properties. Apart from these, roots are used for diabetic carbuncles, migraines, and bronchitis [17]. The existing experiment design revealed phytoconstituents such as Cyclotricuspidoside A, Cyclotricuspidoside C, *α*-spinasterol, Stigmast-7-en-3*β*-ol, 3-o-*β*-D-glucopyranoside, and Glyceryl-1-palmitate [18]. The current experiment design suggested cytotoxic Cucurbitacins activity reported from the fruits [19].

*Amorphophallus campanulatus* is commonly known as Elephant foot yam. Generally, it is a herbaceous, longstanding plant, and it can be 0.75 m in height and belongs to the family of *Arecaceae* [20]. Traditionally, it has been used for several purposes for example tumors, spleen enlargements, asthma, and rheumatism. The plant's tuberous roots have also been found to have tonic, stomach, and appetizing properties. Infusion of leaf stalks is useful in bites of poisonous insects [21]. The recent study evaluated phytoconstituents such as Stigmasterol; *β*-Sitosterol; Campesterol; 1,3,5-Benzenetriol; Vitamin E acetate; and Squalene. The latest study reported anthelminthic activity from tuber [22].

Although these three plants have several important traditional uses, no scientific research has been carried out to determine their activity against diabetes and oxidative stress; that is why the present study has aimed to evaluate those three medicinal plants' antidiabetic and antioxidant activity through experimental (*in vitro* study) and computational techniques (molecular docking, ADME/T study, and PASS predictions).

## 2. Materials and Methods

### 2.1. Plant Collection and Identification

Leaves of *Molineria capitulata*, *Trichosanthes tricuspidata*, and *Amorphophallus campanulatus* were obtained from the hilly area of Kaptai, Chittagong, Bangladesh, on August 16, 2019. The plants were identified by a renowned taxonomist from Bangladesh.

### 2.2. Preparation of Plant Material and Extract Preparation

Normally each of the plant materials was collected in fresh condition. Then, the leaves dried under shade and ground for 10 days. The materials were ground to obtain coarse powder and finally preserved in an airtight container. The leaves powder (100 gm) was soaked in 500 ml of ethanol (*M*. *capitulata*) and methanol (*A*. *campanulatus* and *T*. *tricuspidata*) for 7 days at room temperature (25.0 ± 0.5)°C. Then, the solvent was refined and evaporated extra liquid portion through water bath to leave a viscous mass. Furthermore, it is placed at room temperature for a while for getting dried extract.

### 2.3. In Vitro Study: In Vitro *α*-Amylase Inhibition Assay for Antidiabetic Activity

The assay was acted in an act following based on the starch-iodine test [23]. In brief, 1 ml of plant extract of different concentrations (1000−125 *μ*g/ml) was added to 1 ml of Na₃PO₄ buffer (pH 6.9 full of 6 mmol NaCl) containing 0.04 units of *α*-amylase solution. The mixture was incubated at 37°C for 10 min to complete the reaction. Then, 1 ml soluble starch (1% w/v) was added to each concentration and again incubated at 37°C for 15 min. Again 1 M HCl (40 *μ*L) was added, then followed by the addition of 200 *μ*l of iodine reagent. The absorbance was estimated at 620 nm in a UV-spectrophotometer.

### 2.4. In Vitro Study: Antioxidant Activity

#### 2.4.1. Qualitative Estimation of 2,2-Diphenyl-l-Picryl-Hydrazyl-Hydrate (DPPH)

The free radical scavenging performance of samples was carried out in terms of hydrogen donating or radical-scavenging ability using the stable radical DPPH [24]. In short, a test sample or standard 0.1 ml at different concentrations (500 to 15.625 *μ*g/ml) was added to 3 ml of a 0.004% methanol solution of DPPH in a test tube. The mixture was incubated for 30 min at room temperature to complete the reaction. Then, the absorbance was measured at 517 nm by using a UV-visible spectrophotometer against a blank. The % inhibition was carried out by the following equation ((*A*0 − *A*1)/*A*0) × 100, where *A*0 denotes the absorbance of the control and *A*1 denotes the test sample or standard absorption.

#### 2.4.2. Qualitative Estimation of Reducing Power

The transformation of Fe^3+^ to Fe^2+^ can be visualized by the determination of reducing power [25]. In brief, 1 ml of the test sample or standard (500 to 15.625 *μ*g/ml) was blended with 0.2 M phosphate buffer 2.5 ml (pH 6.6) along with potassium ferricyanide 2.5 ml, (1% w/v). The blend was incubated to complete the reaction at 50°C and the duration was 20 min. Furthermore, 2.5 ml of trichloroacetic acid (TCA) (10% v/v) was added to the mixture and then centrifuged for 10 min at 3000 rpm. An equal amount (2.5 ml) supernatant layer of the solution and distilled water was mixed after that 0.5 ml of FeC1_3_ (0.1% w/v) was added. Then, the absorbance was evaluated at 700 nm by using a UV-visible spectrophotometer against blank.

#### 2.4.3. Quantitative Estimation of TPC

Applying the Folin–Ciocalteu reagent to the mixture, the quantity of TPC was carried out [26]. In brief, 0.5 ml of standard/test sample (1.00 mg/ml) at different concentrations (500 to 15.625 *μ*g/ml), 2.5 ml of the Folin–Ciocalteu reagent (FCR) was added. After that within 0.5 to 8 min, 2 ml of Na_2_CO_3_ (7.5%) was added. The mixture was incubated for 5 min to carry out the reaction at 50°C and then cooled. Then, the absorbance was measured at 760 nm by utilizing the UV-visible spectrophotometer opposite to the blank.

#### 2.4.4. Quantitative Estimation of TFC

By executing an aluminum colorimetric assay, the quantity of TFC was evaluated [26]. About 1.00 ml of test/standard (100 to 12.5 *μ*g/ml) was blended with 3.00 ml of methanol (CH_3_OH), 0.2 ml of 10% AlCl_3_, 0.2 ml of 1 M potassium acetate, and 5.6 ml of distilled water. The mixture was incubated at room temperature (25°C) to carry out the reaction for half an hour. Then, the absorbance was estimated at 420 nm with the help of a UV-visible spectrophotometer versus blank.

### 2.5. In Silico Study

#### 2.5.1. Chemical Compounds and Proteins

Selective compounds were downloaded from PubChem [27] as a 2D SDF file for comparative docking investigation against a standard candidate. The compounds were ascorbic acid, gallic acid, and acarbose, selected from the previous investigation [14, 18, 22]. Then, relevant proteins were taken as PDB files from Protein Data Bank [28]; these proteins were pancreatic *α*-amylase [PDB: 3BAJ] and uricase [PDB: 1R4U].

#### 2.5.2. Ligand and Protein Preparation

The selected compounds and standard drugs were prepared to utilize LigPrep wizard, a bioinformatics tool included in Maestro (Schrödinger software v 11.1). The compounds were fixed as project-table files; in addition, the other parameters were kept in the default set-up. Thereafter, importing the anticipated protein as PDB-format as well as performing preprocess job by dint of Protein Preparation Wizard. Subsequently, the protein molecules were prepared by eliminating the water molecules (<3 H-bonds to nonwaters). The force field is fixed at OPLS3 during the minimization process. Furthermore, supplementary parameters were kept in the default situation. Afterward, the receptor grid was generated by the use of the Receptor-Grid Generation Wizard. PockDrug [28], an online tool, was used to pick the best docking goal in keeping with the highest druggability probability value. The *X*, *Y*, and *Z* axis has been kept within 6 to 14 in case of the advanced setting of a target site.

#### 2.5.3. Glide Standard Precision (SP) Ligand Docking

The flexible-docking was directed between protein molecules and legends to recognize possible biological mechanisms completed by Maestro (v 11.1). The docking interaction was executed utilizing the Ligand-Docking Wizard to promote the ligands for docking based on binding strength. Throughout this operation, all factors were preserved in the default function, and to get the best output, the docking job was being executed for several times. Lastly, a docking spreadsheet was gathered for additional data analysis. For a better understanding of the docking relations, 2D as well as 3D figures were occupied by a molecular imagining tool (Discovery studio-v 4.1).

#### 2.5.4. ADME, Toxicological Property, and PASS Prediction Analysis

Diverse biokinetics properties in addition to toxicological properties such as absorption, distribution, metabolism, excretion, and toxicity (ADME/T) are measured during drug development. The ADME and toxicological properties defined above are estimated by [29] and AdmetSAR [30], respectively, which are online bioinformatics depositories. Depending on Lipinski's and Veber's rules, subsequent parameters were audited to guess drug-likeness behaviors, for instance, molecular weight (MW), hydrogen-bond acceptor (HBA), hydrogen-bond donor (HBD), lipophilicity (logP), number of the rotatable-bond (NRB), and topological polar surface area (TPSA). Then, again rat-acute toxicity, acute-oral toxicity, as-toxicity, and carcinogenic were measured through assessment of toxicity. PASS Online [31], an online bioinformatics platform, was used to accomplish biological prediction.

### 2.6. Statistical Analysis

All assays were conducted in triplicate and results are presented as means ± SEM (standard errors of means). The differences among different test groups were analyzed by one-way analysis of variance (ANOVA) followed by Tukey's honestly significant difference post hoc test with *α* = 0.01. All of the data analyses were performed on GraphPad Prism version 8.0.2 for Windows (GraphPad Software version 8.0.2, San Diego, California, USA).

## 3. Results

### 3.1. In Vitro *α*-Amylase Inhibition Assay for Antidiabetic Activity

**Figure 1:**
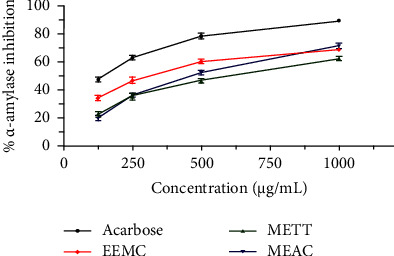
Data are expressed in mean ± SEM (*n* = 3); the standard denotes acarbose for *α*-amylase inhibition assay, respectively. EEMC: ethanol extract of *M*. *capitulata*; METT: methanol extract of *T*. *tricuspidata*; MEAC: methanol extract of *A*. *campanulatus*.

In this *α*-amylase inhibition, assay all three plant extracts demonstrated promising antidiabetic bioactivity comparison with acarbose as a potential antidiabetic drug described in Table 1 and Figure 1. The percentage of *α*-amylase inhibition increased with the increase of concentration density. Between 150 and 875 *μ*g/mL concentrations, the EEMC showed the highest inhibition rate of the remaining two samples (METT and MEAC) with the IC_50_ value of 300.9 ± 3.38 *μ*g/mL. However, the IC_50_ value of the *α*-amylase inhibition assay was 133.3 ± 0.82 *μ*g/mL. The order for percentage of *α*-amylase inhibition was as follows: acarbose > EEMC > MEAC > METT at 1000 *μ*g/mL conc.

### 3.2. Antioxidant Activity

#### 3.2.1. Quantitative Estimation of DPPH

**Figure 2:**
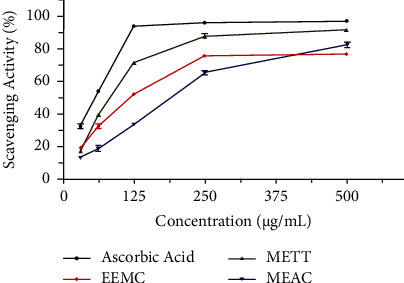
Effect of different extracts on DPPH scavenging activity. Data are expressed in mean ± SEM (*n* = 3); EEMC: ethanol extract of *M*. *capitulata*; METT: methanol extract of *T*. *tricuspidata*; MEAC: methanol extract of *A*. *campanulatus*.

Utilizing the DPPH free radical scavenging qualitative assay, the antioxidant activity of EEMC, METT, and MEAC was studied compared to the standard candidate (ascorbic acid) summarized in Table 2 and Figure 2. Among all three samples extracted, the METT significantly showed the highest scavenging activity at 500 *μ*g/mL concentration (*P* < 0.01) with concentration-dependent tendency, and the minimum IC_50_ value was 81.88 ± 0.99 *μ*g/mL while the IC_50_ value was 116.7 ± 2.21 and 162 ± 1.7 *μ*g/mL for EEMC and MEAC, respectively. However, the minimum inhibitory concentration value of the standard drug was 45.43 ± 0.75 *μ*g/mL.

#### 3.2.2. Quantitative Estimation Reducing Power Activity

**Figure 3:**
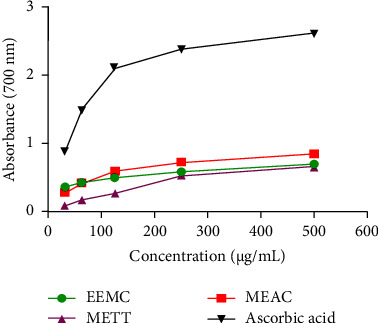
Effect of different extracts on reducing power activity. Data are expressed in mean ± SEM (*n* = 3); EEMC: ethanol extract of *M*. *capitulata*; METT: methanol extract of *T*. *tricuspidata*; MEAC: methanol extract of *A*. *campanulatus*.

By maintaining a concentration-dependent manner EEMC, METT, and MEAC moderately showed a reducing power effect compared to ascorbic acid at 700 nm absorbance represented in Figure 3. The MEAC showed maximum absorbance of 0.840 at 500 *μ*g/mL conc. while ascorbic acid showed 2.587 at the same concentration. The sequence for reducing power activity was as followed: ascorbic acid > MEAC > EEMC > METT at 500 *μ*g/mL concentration.

#### 3.2.3. Quantitative Estimation of TPC and TFC

The quantitative investigation of antioxidant-related phytochemicals with TPC as well as TFC of EEMC, METT, and MEAC are summarized in Table 3. The METT exhibited the highest total phenol content (107.50 ± 1.58 mg GAE/g), whereas EEMC and METT showed 57.92 ± 1.72 and 65.78 ± 1.06 mg GAE/g phenolic contents, respectively. Then, again EEMC exhibited the highest total flavonoid content (142.7 ± 2.86 mg GAE/g) while EEMC and METT showed 116.60 ± 1.67 and 70.27 ± 2.06 mg GAE/g flavonoid contents, respectively.

### 3.3. In Silico Study

#### 3.3.1. Docking Study for *α*-Amylase Inhibition

**Figure 4:**
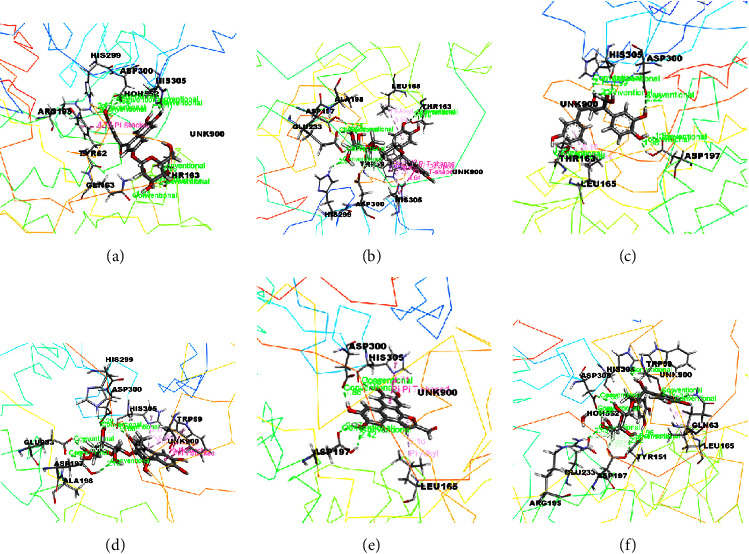
The best-ranked pose of the major compounds of EEMC: (a) Crassifoside A, (b) Breviscaside A, (c) Crassifogenin C, (d) Crassifoside D, (e) curcapital, and (f) Isocurculigine in the binding pocket of *α*-amylase [PDB ID: 3BAJ)]for antidiabetic activity.

**Figure 5:**
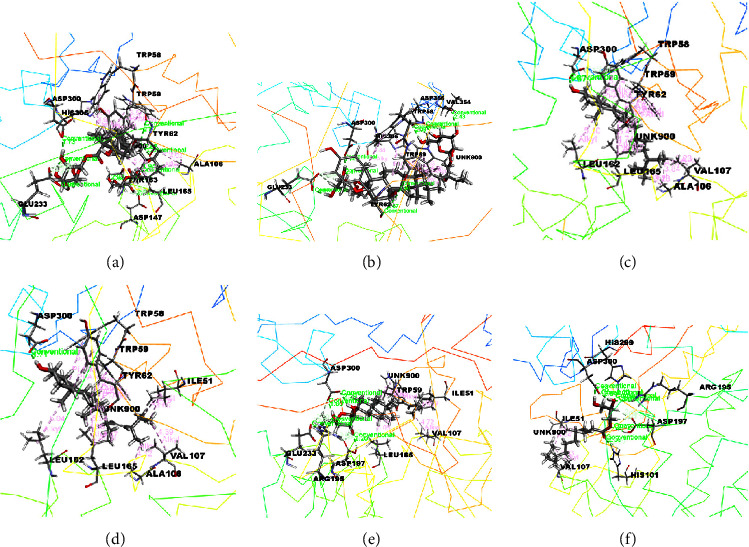
The best-ranked pose of the major compounds of METT: (a) Cyclotricuspidoside A, (b) Cyclotricuspidoside C, (c) Stigmast-7-en-3*β*-ol, (d) *α*-spinasterol, (e) 3-o-*β*-D-glucopyranoside, and (f) glyceryl 1 palmitate in the binding pocket of *α*-amylase [PDB ID: 3BAJ] for antidiabetic activity.

**Figure 6:**
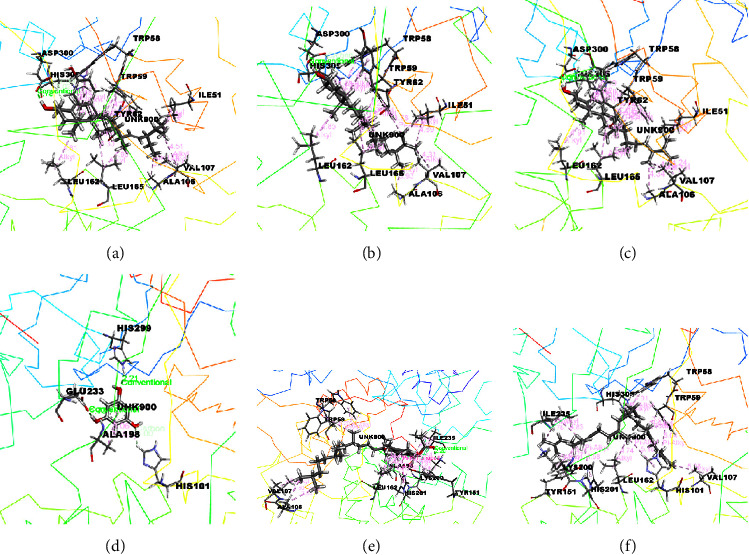
The best-ranked pose of the major compounds of MEAC: (a) Stigmasterol, (b) *β*-Sitosterol, (c) Campesterol, (d) 1,3,5-Benzenetriol, (e) Vitamin E acetate, and (f) Squalene in the binding pocket of *α*-amylase (PDB ID: 3BAJ) for antidiabetic activity.

The docking results associated with the antidiabetic activity are informed in Table 4, and the compounds of EEMC, METT, and MEAC with the maximum docking score are demonstrated in Figures 4–6. Based on bioactivity protein molecules named pancreatic *α*-amylase [PDB: 3BAJ] were used for respective molecular simulation with 18 selective compounds including the following parameters as docking score, glide model, and glide energy compared with standard drug Acarbose. In EEMC, Breviscaside A has shown the best docking affinity (−6.06 kcal/mol). Cyclotricuspidoside A exposed the maximum docking score not only in METT, but also in 18 compounds (−7.19 kcal/mol) which is relatively close to Acarbose (−7.37 kcal/mol). Cyclotricuspidoside A interacted with ASP A: 147, ASP A: 300, and GLU A: 233 via H-bond and one van der Waals bond with THR A: 163. Moreover, MEAC Stigmasterol and *β*-Sitosterol showed top docking affinity, −6.08 and −6.06 kcal/mol, respectively.

#### 3.3.2. Docking Study for Antioxidant Activity

**Figure 7:**
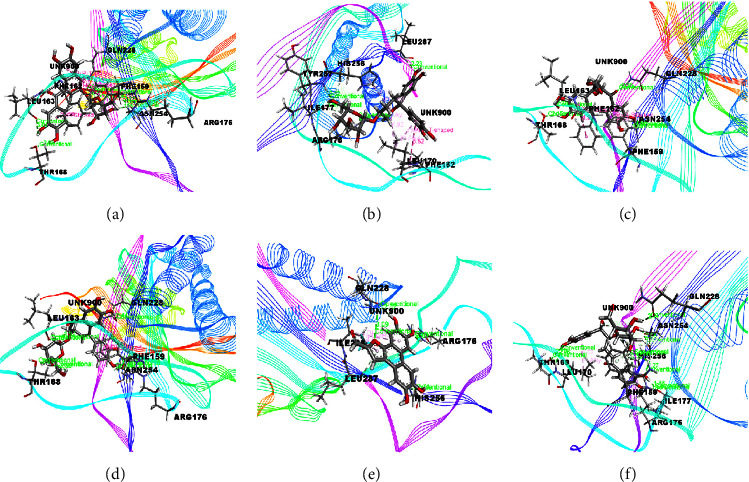
The best-ranked pose of the major compounds of EEMC: (a) Crassifoside A, (b) Breviscaside A, (c) Crassifogenin C, (d) Crassifoside D, (e) Curcapital, and (f) Isocurculigine in the binding pocket of Uricase (PDB ID: 1R4U) for antioxidant activity.

**Figure 8:**
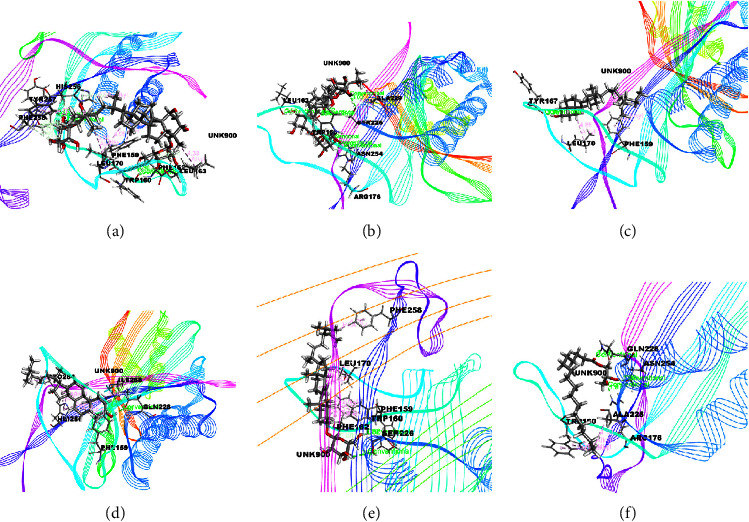
The best-ranked pose of the major compounds of METT: (a) Cyclotricuspidoside A, (b) Cyclotricuspidoside C, (c) Stigmast-7-en-3*β*-ol, (d) *α*-Spinasterol, (e) 3-o-*β*-D-Glucopyranoside, and (f) Glyceryl 1 palmitate in the binding pocket of Uricase (PDB ID: 1R4U) for antioxidant activity.

**Figure 9:**
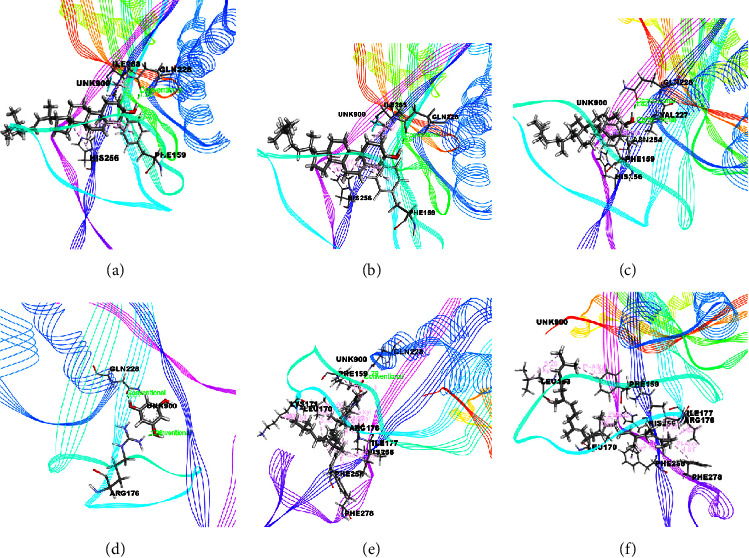
The best-ranked pose of the major compounds of MEAC: (a) Stigmasterol, (b) *β*-Sitosterol, (c) Campesterol, (d) 1,3,5-Benzenetriol, (e) Vitamin E acetate, and (f) Squalene in the binding pocket of Uricase [PDB ID: 1R4U] for antioxidant activity.

The docking results associated with antioxidant activities are shown in Table 5 and the compounds of EEMC, METT, and MEAC with the maximum docking score are demonstrated in Figures 7–9. Based on bioactivity, a protein molecule named Uricase [PDB: 1R4U] was used for respective molecular simulation with 18 selective compounds containing the following parameters as docking score, glide model, and glide energy compared with three standard drugs ascorbic acid, gallic acid, and quercetin. The docking scores are ascorbic acid (−4.52 kcal/mol), gallic acid (−5.07 kcal/mol), and quercetin (−5.15 kcal/mol). In EEMC, isocurculigine (−5.73 kcal/mol) showed the best score which is better than the three standards. Cyclotricuspidoside A (−6.06 kcal/mol) exhibited the top score in METT. Furthermore, 1,3,5-Benzenetriol, a compound of MEAC, showed the strongest docking score among 18 compounds as well as more than all standards (−6.22 kcal/mol). 1,3,5-Benzenetriol interacted with ARG A: 170 and GLN A: 228 via two H-bond and one van der Waals bond with VAL A: 227. In addition to these, Crassifoside A (−5.35 kcal/mol), Crassifogenin C (−5.34 kcal/mol), Crassifoside D (−5.26 kcal/mol), Crassifoside D (−5.26 kcal/mol), and Cyclotricuspidoside C (−5.24 kcal/mol) showed more docking affinity than the three standards.

#### 3.3.3. ADME Analysis

As reported by Lipinski's rule, two compounds of METT (Cyclotricuspidoside A and Cyclotricuspidoside C) violate three of Lipinski's rules. Moreover, no other compounds from other plants violate Lipinski's rule. On the other hand, according to Veber's rules, seven compounds from different plants violated one rule of Veber, among them Cyclotricuspidoside A and Cyclotricuspidoside C (from METT) violate two conditions (Table 6).

#### 3.3.4. Toxicity

In toxicity analysis, 18 compounds from three plants, among which Cyclotricuspidoside A and Cyclotricuspidoside C compounds of METT, exhibited the highest acute toxicity (Table 7).

#### 3.3.5. Pass Predictions

Pass investigation demonstrated hypothetical pharmacological action of each of the main compounds within MEAC, METT, and MEAC. We appraised different organic activities for each compound and considered the values of Pa > Pi and Pa > 7. This assumption proposes several immense compounds studied, such as antidiabetic, antioxidant, insulin promoter, free radical scavenger, and *α*-glucosidase inhibitor activities which are related to our present study. The guessed pharmacological activities of all (6 compounds of each plant) main compounds are presented in Tables 8 and 9.

## 4. Discussion

Currently, wild plants are regarded to have high dietary values because of superior fiber, polyphenol substances, and higher antioxidant capacity than cultured plants. Furthermore, numerous plants have exhibited to be fantastic efficacy in continual diseases for example cardiovascular diseases and diabetes. Quite a bit of this information has been orally passed from age-to-age which has prompted the advancement of the typical health care system, practiced in different nations of the world [32, 33]. Instinctively, inhibitors of *α*-amylase by food-grade natural sources deliver an appealing remedial way to deal with the treatment of postprandial hyperglycemia through diminishing glucose discharge from starch, which might be possibly valuable in the treatment of diabetes mellitus and weight problems [8]. On the other hand, the cell's protection components can be either endogenous or exogenous; for this, the indispensable section is antioxidant [34]. In this study, plotted three individual (EEMC, MEAC, and METT) three plant extracts for *in vitro* inhibition of *α*-amylase and antioxidant activities along with *in silico* study (molecular docking, ADME/T, PASS predictions, and DFT activities) have been exhibited.

In the present study, inhibition of *α*-amylase of three plants (EEMC, MEAC, and METT) explores the significant effect, but EEMC demonstrates the highest impact among the other three extracts as compared to a reference to the standard drug (acarbose) additionally which also is statistically significant. This inhibition firmly demonstrated the presence of some phytoconstituents; these constituents are responsible for this inhibition and may occur following compounds that are responsible for these activities such as saponin, steroid, and terpenoid [35]. Contemporary investigations have stated the enzyme inhibitory activities of plant phenolics with a solid inhibitory impact on *α*-glucosidase, but a gentle impact on *α*-amylase, therefore proposing its utilization for the cure and the executives of diabetes [36]. The *α*-amylase and *α*-glucosidase are alluded to as promising therapeutic effect in diabetes which inhibits and delay the action of starch consumption enzymes [37].

Moreover, the antioxidant efficacy of separate three plant extracts (EEMC, MEAC, and METT) was revealed based on the qualitative DPPH free radical scavenging activity and reducing power capacity assay and quantitative total TPC and TFC. Furthermore, this investigation showed among three different plants extract; METT possesses a potent antioxidant effect as compared to thestandard (ascorbic acid). The antioxidant mechanism privileges the reduction formation of the hydroxyl radicals throughout lipid peroxidation. The transition metallic ion Fe^2+^ has the potential to uproot a single electron by way of the distinctive feature of which it may disable the placing and extension of numerous radical responses [38]. Apart from this, the study also reveals reducing power activity of EEMC, MEAC, and METT has increased in a concentration-dependent manner. The presence of polyphenolic compounds (flavonoids, phenolic acids, and tannins) may be responsible for reducing the power activity of plants which also indicates the strong potentiality of the antioxidant activity [39].

Furthermore, molecular docking is one of the incredible assets to exploring the dynamic site of the protein and additionally comprehending and clarifying the binding associations among the ligands and desired protein [40]. In the molecular study of *α*-amylase inhibition study, we have selected 18 (each plant represents the 6 compounds) major compounds of EEMC, METT, and MEAC. We interact compounds individually with the targeted protein pancreatic *α*-amylase [PDB: 3BAJ]; in a comparison study, we noticed that the compounds of METT (Cyclotricuspidoside A and Cyclotricuspidoside C) had displayed the highest docking score, which is almost closed to the standard reference drug acarbose. Along with this, the Crassifogenin C and Breviscaside A compounds of METT showed the highest docking score than the Stigmasterol and *β*-Sitosterol compounds of MEAC. Docking score, Glide Emodel, and Glide energy was considered.

Subsequently, *in silico* antioxidant molecular docking study of 18 compounds of EEMC, METT, and MEAC (each plant of 6 compounds) was carried out through interaction with the targeted protein Uricase [PDB: 1R4U]. In this study, we evaluated the compounds of METT (Cyclotricuspidoside A and Cyclotricuspidoside C) exhibited prominent results as compared to other compounds which are higher than the standard, on the other hand EEMC (Isocurculigine and Crassifoside A) and MEAC (1,3,5-Benzenetriol and Campesterol) also possess potent outcome as compared to the standard which is also most closed to the standard. However, we considered the Docking Score, Glide Emodel, and Glide Energy. It could have the function of rival antioxidant effect on the protein, those facts are in complete agreement with the associated good docking rating and binding affinity.

Furthermore, for 18 compounds, we established their pharmacokinetic properties' physiochemical aspects, and drug-likeness, through ADME analysis, which is an online server basis program. We follow the two rules, one is Lipinski's rule and the other is Veber's rule; according to these rules, Cyclotricuspidoside A and Cyclotricuspidoside C of METT were violets maximum rules; on the other hand, those two compounds did not obey Veber's rules. It is proclaimed that as much as lower molecular weight, higher tendency to dissolving, and the ability of hydrogen bonds to have high permeation ability with favorable absorption rate and bioavailability.

Subsequently, we performed toxicity tests online to find the toxicity properties of EEMC, METT, and MEAC; we considered a few parameters such as Ames toxicity, carcinogens, and acute oral toxicity. We also noticed the LD50, but among the 18 compounds from three plants, only two compounds (Cyclotricuspidoside A and Cyclotricuspidoside C) possessed the highest LD50 value.

Moreover, for the prediction of efficacy of the plant substance activity, we analyzed by prediction of activity spectra for substances (PASS), which assessed the biological activity of prediction. The outcome proposes many activities, among them we ascertained 18 compounds of EEMC, METT, and MEAC possible activity values, which lays under the Pa range 0.123 to 0.780.

## 5. Conclusions

The output may indicate that the EEMC, METT, and MEAC possess profound *α*-amylase inhibition and antioxidant activities. Therefore, the present study proposes a scientific basis for implementing this plant to manage various illnesses. However, this is only an initial study. Further in-depth, precise molecular studies are warranted in *in silico* analysis to reveal that these compounds will be the source of the new biological activity.

## Figures and Tables

**Table 1:** The IC_50_ values for the *α*-amylase inhibition of EEMC, METT, and MEAC.

Sample	*α*-amylase inhibition IC_50_(*μ*g/mL)
EEMC	300.9 ± 3.38^b^
METT	547.7 ± 3.26^d^
MEAC	431.6 ± 2.26^c^
Standard	133.3 ± 0.82^a^

Data are expressed in mean ± SEM (standard errors of mean); different superscript letters (a, b, c, and d) in a column indicate significant difference at *p* < 0.01; EEMC: ethanol extract of *M*. *capitulata*; METT: methanol extract of *T*. *tricuspidata*; MEAC: methanol extract of *A*. *campanulatus*; standard denotes acarbose for *α*-amylase inhibition assay, respectively.

**Table 2:** The IC_50_ values for the DPPH free radical scavenging activities of the extracts.

Sample	DPPH assay IC_50_ (*μ*g/mL)
EEMC	116.7 ± 2.21^c^
METT	81.88 ± 0.99^b^
MEAC	162 ± 1.7^d^
Standard	45.43 ± 0.75^a^

Data are expressed in mean ± SEM (standard errors of mean); different superscript letters (a, b, c, and d) in a column indicate significant difference at *p* < 0.01; EEMC: ethanol extract of *M*. *capitulata*; METT: methanol extract of *T*. *tricuspidata*; MEAC: methanol extract of *A*. *campanulatus*.

**Table 3:** Quantitative total phenolic and flavonoid content of EEMC, METT, and MEAC.

Sample	TPC (mg GAE/g)	TFC (mg QE/g)
EEMC	57.92 ± 1.72^b^	116.60 ± 1.67^b^
METT	107.50 ± 1.58^a^	70.27 ± 2.06^c^
MEAC	65.78 ± 1.06^b^	142.7 ± 2.86^a^

Data are expressed in mean ± SEM (standard errors of mean); different superscript letters (a, b, c, and d) in a column indicate significant difference at *p* < 0.01; EEMC: ethanol extract of *M*. *capitulata*; METT: methanol extract of *T*. *tricuspidata*; MEAC: methanol extract of *A*. *campanulatus*; GA: gallic acid; QE: quercetin.

**Table 4:** Molecular docking study of EEMC, METT, and MEAC against *α*-amylase (PDB ID: 3BAJ) for antidiabetic activity.

Sample	Compound	Docking score	Glide Emodel	Glide energy
EEMC	Crassifoside A	−6.03	−67.87	−50.98
	Breviscaside A	−6.06	−69.67	−54.05
	Crassifogenin C	−6.03	−67.87	−50.98
	Crassifoside D	−5.92	−65.15	−51.27
	Curcapital	−5.34	−51.50	−38.20
	Isocurculigine	−5.71	−76.74	−59.01

METT	Cyclotricuspidoside A	−7.19	−86.64	−60.86
	Cyclotricuspidoside C	−6.89	−87.29	−60.02
	Stigmast-7-en-3*β*-ol	−5.56	−50.89	−37.71
	*α*-Spinasterol	−6.18	−52.09	−37.94
	3-o-*β*-D-glucopyranoside	−5.26	−58.87	−50.47
	Glyceryl 1 palmitate	−5.06	−54.98	−45.95

MEAC	Stigmasterol	−6.08	−51.93	−37.41
	*β*-sitosterol	−6.06	−49.61	−49.61
	Campesterol	−5.89	−48.07	−35.58
	1,3,5-Benzenetriol	−5.25	−35.03	−25.74
	Vitamin E acetate	−4.43	−56.71	−47.62
	Squalene	−3.15	−47.09	−42.01

Standard	Acarbose	−7.37	−81.25	−59.51

EEMC: ethanol extract of *M*. *capitulata*; METT: methanol extract of *T*. *tricuspidata*; MEAC: methanol extract of *A*. *campanulatus*.

**Table 5:** Molecular docking study of EEMC, METT, and MEAC against Uricase (PDB ID: 1R4U) for antioxidant activity.

Sample	Compound	Docking score	Glide Emodel	Glide energy
EEMC	Crassifoside A	−5.35	−59.52	−47.16
	Breviscaside A	−4.16	−53.09	−43.10
	Crassifogenin C	−5.34	−53.21	−41.99
	Crassifoside D	−5.26	−55.67	−45.15
	Curcapital	−4.75	−39.26	−30.77
	Isocurculigine	−5.73	−70.01	−55.83

METT	Cyclotricuspidoside A	−6.06	−70.04	−56.64
	Cyclotricuspidoside C	−5.24	−67.78	−53.35
	Stigmast-7-en-3*β*-ol	−2.95	−26.46	−24.87
	*α*-Spinasterol	−4.26	−32.43	−25.42
	3-o-*β*-D-glucopyranoside	−2.74	−33.84	−30.30
	Glyceryl 1 palmitate	−3.02	−36.58	−33.96

MEAC	Stigmasterol	−3.37	−26.59	−22.17
	Beta-sitosterol	−3.44	−27.66	−23.70
	Campesterol	−4.02	−30.20	−24.95
	1,3,5-Benzenetriol	−6.22	−29.66	−21.28
	Vitamin E acetate	−3.59	−41.48	−37.40
	Squalene	−2.40	−32.13	−31.20

Standard	Ascorbic acid	−4.52	−32.91	−25.72
	Gallic acid	−5.07	−34.53	−26.13
	Quercetin	−5.15	−44.42	−34.35

EEMC: ethanol extract of *M*. *capitulata*; METT: methanol extract of *T*. *tricuspidata*; MEAC: methanol extract of *A*. *campanulatus*.

**Table 6:** ADME property prediction of major compounds of EEMC, METT, and MEAC by using Swiss ADME online tool.

Sample	Compound	Lipinski's rules					Lipinski's violations ≤1	Veber's rules	
		MW (g/mol) <500	HBA <10	HBD ≤10	Log *P*	Molar refractivity (40–130)		NRB ≤10	TPSA ≤140
EEMC	Crassifoside A	474.41	11	8	0.10	116.06	2	5	197.37
	Breviscaside A	480.46	11	8	−0.10	115.61	2	5	189.53
	Crassifogenin C	348.30	8	6	0.54	84.81	1	5	150.98
	Crassifoside D	478.45	11	9	−0.62	116.28	2	4	200.53
	Curcapital	310.26	6	4	1.97	84.71	No	1	111.13
	Isocurculigine	496.46	12	9	−0.65	118.34	2	9	217.60

METT	Cyclotricuspidoside A	845.09	16	11	1.08	210.07	3	13	276.52
	Cyclotricuspidoside C	861.02	17	12	0.31	211.24	3	13	296.75
	Stigmast-7-en-3*β*-ol	414.71	1	1	7.18	133.23	1	6	20.23
	*α*-Spinasterol	412.69	1	1	6.88	132.75	No	5	20.23
	3-o-*β*-D-glucopyranoside	574.83	6	4	−2.20	165.14	2	8	99.38
	Glyceryl 1 palmitate	330.50	4	2	4.64	97.06	No	18	66.76

MEAC	Stigmasterol	412.69	1	6	6.96	132.75	No	5	20.23
	*β*-sitosterol	414.71	1	1	7.19	133.23	No	6	20.23
	Campesterol	400.68	1	1	6.90	128.42	No	5	20.23
	1,3,5-Benzenetriol	126.11	3	3	2.50	32.51	No	0	60.69
	Vitamin E acetate	472.74	3	0	8.57	148.75	1	14	35.53
	Squalene	410.72	0	0	9.80	143.48	1	15	0.00

MW: molecular weight; HBA: hydrogen bond acceptor; HBD: hydrogen bond donor; lipophilicity (expressed as Log *P*) and molar refractivity. NRB: number of rotatable bonds; TPSA: topological surface area; EEMC: ethanol extract of *M*. *capitulata*; METT: methanol extract of *T*. *tricuspidata*; MEAC: methanol extract of *A*. *campanulatus*.

**Table 7:** Toxicological property analysis of EEMC, METT, and MEAC.

Sample	Compound	Parameters			
		Ames toxicity	Carcinogens	Acute oral toxicity	Rate of acute toxicity (LD_50_, mol/kg)
EEMC	Crassifoside A	NAT	NC	III	2.5150
	Breviscaside A	NAT	NC	III	2.2683
	Crassifogenin C	NAT	NC	III	2.8380
	Crassifoside D	NAT	NC	III	2.8786
	Curcapital	NAT	NC	III	2.0528
	Isocurculigine	NAT	NC	III	2.1009

METT	Cyclotricuspidoside A	NAT	NC	I	3.6250
	Cyclotricuspidoside C	NAT	NC	I	3.6250
	Stigmast-7-en-3*β*-ol	NAT	NC	III	2.4189
	*α*-Spinasterol	NAT	NC	III	2.4107
	3-o-*β*-D-glucopyranoside	NAT	NC	IV	1.3577
	Glyceryl 1 palmitate	NAT	NC	IV	0.8172

MEAC	Stigmasterol	NAT	NC	I	2.6561
	*β*-sitosterol	NAT	NC	I	2.6561
	Campesterol	NAT	NC	I	2.8078
	1,3,5-Benzenetriol	NAT	NC	III	1.7622
	Vitamin E acetate	NAT	NC	IV	1.5022
	Squalene	NAT	NC	III	1.5057

NAT: not Ames toxicity: NT, not carcinogen; LD: lethal dose; EEMC: ethanol extract of *M*. *capitulata*; METT: methanol extract of *T*. tricuspidata; MEAC: methanol extract of *A*. *campanulatus*.

**Table 8:** Biological activities found for the ethanol extract of *M*. *capitulata* (EEMC) and methanol extracts of *T*. *tricuspidata* (METT) and *A*. *campanulatus* (MEAC) major compounds by PASS online.

Sample	Compound	Biological properties predicted by PASS online	Pa	Pi
EEMC	Crassifoside A	Antidiabetic	0.696	0.006
		Antioxidant	0.620	0.004
		Free radical scavenger	0.772	0.003
	Breviscaside A	Antidiabetic	0.597	0.013
		Alpha glucosidase inhibitor	0.637	0.001
		Antioxidant	0.495	0.007
	Crassifogenin C	Insulin promoter	0.279	0.007
		Antioxidant	0.615	0.168
		Free radical scavenger	0.364	0.021
	Crassifoside D	Antioxidant	0.509	0.006
		Lipid peroxidase inhibitor	0.579	0.010
		Free radical scavenger	0.626	0.005
	Curcapital	Insulin inhibitor	0.483	0.055
		Antioxidant	0.291	0.025
		Free radical scavenger	0.323	0.026
	Isocurculigine	Antidiabetic	0.618	0.011
		Antioxidant	0.539	0.005
		Free radical scavenger	0.669	0.004

METT	Cyclotricuspidoside A	Antidiabetic	0.417	0.039
		Alpha glucosidase inhibitor	0.123	0.018
		Antioxidant	0.386	0.013
	Cyclotricuspidoside C	Antidiabetic	0.362	0.055
		Alpha glucosidase inhibitor	0.124	0.017
		Antioxidant	0.377	0.014
	Stigmast-7-en-3*β*-ol	Alpha glucosidase inhibitor	0.069	0.064
		Insulin promoter	0.547	0.021
		Antioxidant	0.172	0.077
	*α*-Spinasterol	Alpha glucosidase inhibitor	0.069	0.064
		Insulin promoter	0.527	0.024
		Antioxidant	0.208	0.051
	3-o-*β*-D-glucopyranoside	Antioxidant	0.373	0.015
		Lipid peroxidase inhibitor	0.305	0.062
		Free radical scavenger	0.317	0.027
	Glyceryl 1 palmitate	Alpha glucosidase inhibitor	0.193	0.005
		Insulin promoter	0.444	0.044
		Antioxidant	0.276	0.028

MEAC	Stigmasterol	Insulin promoter	0.347	0.095
		Lipid peroxidase inhibitor	0.305	0.062
		Antioxidant	0.215	0.048
	*β*-sitosterol	Insulin promoter	0.361	0.085
		Antioxidant	0.178	0.072
		Lipid peroxidase inhibitor	0.237	0.101
	Campesterol	Insulin promoter	0.332	0.107
		Antioxidant	0.182	0.068
		Lipid peroxidase inhibitor	0.273	0.077
	1,3,5-Benzenetriol	Antidiabetic	0.618	0.011
		Alpha glucosidase inhibitor	0.418	0.002
		Free radical scavenger	0.669	0.004
	Vitamin E acetate	Antidiabetic symptomatic	0.510	0.007
		Antioxidant	0.956	0.002
		Free radical scavenger	0.780	0.003
	Squalene	Antioxidant	0.657	0.004
		Lipid peroxidase inhibitor	0.601	0.009
		Free radical scavenger	0.456	0.013

Pa = probable activity; Pi = probable inactivity.

**Table 9:** Global reactivity descriptor values of selective isolated compounds from three different plants.

Derivatives	*I* (eV)	*A* (eV)	*η*	*S*	*μ*	*χ*	*ώ*
Crassifogenin C	5.77263	1.93854	1.91704	0.52164	−3.85558	3.85558	7.43276
Crassifoside A	5.74351	1.95215	1.89568	0.52751	−3.84783	3.84783	7.40289
Stigmasterol	6.20229	−0.69770	3.45000	0.28986	−2.75230	2.75230	3.78757
*β*-Sitosterol	6.20202	−0.76001	3.48102	0.28727	−2.72100	2.72100	3.70193
*α*-Spinasterol	6.14080	−0.64600	3.39340	0.29469	−2.74740	2.74740	3.77410
Stigmassterol-7-en-3*β*-ol	6.14052	−0.73008	3.43530	0.29110	−2.70522	2.70522	3.65911

*I* = ionisation potential; *A* = electron affinity; *η* = global hardness; *S* = global softness; *μ* = chemical potential; *χ* = electronegativity; *ώ* = electrophilicity index.

## Data Availability

All data used to support the findings of this study are included within the article.
